# Discharge communication practices in pediatric emergency care: a systematic review and narrative synthesis

**DOI:** 10.1186/s13643-019-0995-7

**Published:** 2019-04-03

**Authors:** Janet A. Curran, Allyson J. Gallant, Roger Zemek, Amanda S. Newton, Mona Jabbour, Jill Chorney, Andrea Murphy, Lisa Hartling, Kate MacWilliams, Amy Plint, Shannon MacPhee, Andrea Bishop, Samuel G. Campbell

**Affiliations:** 1 School of Nursing, Dalhousie University, 5869 University Ave., PO Box 15000, Halifax, NS B3H 4R2 Canada; 2 Department of Pediatrics, Division of Emergency Medicine, Children’s Hospital of Eastern Ontario, 401 Smyth Rd, Ottawa, ON K1H 8L1 Canada; 3 Department of Pediatrics, Faculty of Medicine & Dentistry, University of Alberta, 11405-87 Avenue, Edmonton, AB T6G 1C9 Canada; 4 IWK Health Center, 5850/5980 University Avenue, PO Box 9700, Halifax, NS B3K 6R8 Canada; 5 College of Pharmacy, Dalhousie University, 5869 University Avenue, PO Box 15000, Halifax, NS B3H 4R2 Canada; 6 Charles V. Keating Emergency and Trauma Centre, QEII Health Sciences Centre, 1796 Summer St, Halifax, NS B3H 3A7 Canada

**Keywords:** Discharge communication, Pediatric emergency care, Caregivers, Systematic review, Narrative synthesis, Integrated knowledge translation

## Abstract

**Background:**

The majority of children receiving care in the emergency department (ED) are discharged home, making discharge communication a key component of quality emergency care. Parents must have the knowledge and skills to effectively manage their child’s ongoing care at home. Parental fatigue and stress, health literacy, and the fragmented nature of communication in the ED setting may contribute to suboptimal parent comprehension of discharge instructions and inappropriate ED return visits. The aim of this study was to examine how and why discharge communication works in a pediatric ED context and develop recommendations for practice, policy, and research.

**Methods:**

We systematically reviewed the published and gray literature. We searched electronic databases CINAHL, Medline, and Embase up to July 2017. Policies guiding discharge communication were also sought from pediatric emergency networks in Canada, USA, Australia, and the UK. Eligible studies included children less than 19 years of age with a focus on discharge communication in the ED as the primary objective. Included studies were appraised using relevant Joanna Briggs Institute (JBI) checklists. Textual summaries, content analysis, and conceptual mapping assisted with exploring relationships within and between data. We implemented an integrated knowledge translation approach to strengthen the relevancy of our research questions and assist with summarizing our findings.

**Results:**

A total of 5095 studies were identified in the initial search, with 75 articles included in the final review. Included studies focused on a range of illness presentations and employed a variety of strategies to deliver discharge instructions. Education was the most common intervention and the majority of studies targeted parent knowledge or behavior. Few interventions attempted to change healthcare provider knowledge or behavior. Assessing barriers to implementation, identifying relevant ED contextual factors, and understanding provider and patient attitudes and beliefs about discharge communication were identified as important factors for improving discharge communication practice.

**Conclusion:**

Existing literature examining discharge communication in pediatric emergency care varies widely. A theory-based approach to intervention design is needed to improve our understanding regarding discharge communication practice**.** Strengthening discharge communication in a pediatric emergency context presents a significant opportunity for improving parent comprehension and health outcomes for children.

**Systematic review registration:**

PROSPERO registration number: CRD42014007106.

**Electronic supplementary material:**

The online version of this article (10.1186/s13643-019-0995-7) contains supplementary material, which is available to authorized users.

## Background

The discharge process in a pediatric emergency department (ED) can introduce vulnerability for parents and caregivers. Attention to this phenomenon is critical given that following a visit to the ED the majority of children are discharged home under the care of their parents [1, 2]. Ideally, parents should depart the ED with the necessary knowledge and skills to effectively manage their children’s care at home. However, following ED discharge, many caregivers and patients are unable to specify their diagnosis, list medications they received, outline post-ED care, or identify when to seek further medical attention [3–5]. The discharge process should include communication of important information about the child’s illness, verification of comprehension, and tailoring of the discharge instructions to address areas of misunderstanding [6]. Yet, this is not always the parents’ experience, and evidence supports that poor quality ED discharge communication can impact subsequent health care utilization, including unscheduled return visits to the ED [7, 8]. Comprehension of discharge communication has been shown to be an important factor in promoting adherence to discharge instructions and preventing unnecessary return visits; however, comprehension is rarely assessed in practice [9]. A number of factors are known to impact comprehension including quality of the communication provided [10], health literacy, numeracy, and reading ability [11–13].

A number of recent reviews have explored discharge communication, including a recent systematic review to establish the cost-effectiveness of implementing electronic discharge communication to support the transition between acute and community care [14]. Another review focused on the parent management of inpatient and ED discharge instructions [15]. We conducted a systematic review and narrative synthesis of the discharge communication literature to gain a better understanding of how and why discharge communication functions in a pediatric ED context and to inform the development of recommendations for future research, policy, and practice change. Our review addressed the following questions: (1) What types of interventions, processes, and policies have been examined regarding discharge communication in a pediatric ED context and (2) How does the discharge process impact parent, patient, and provider outcomes?

## Methods

### Study design

We conducted a systematic review with narrative synthesis following methods outlined by Popay et al. [16]. This type of review explores relationships within and between studies [17]. Techniques are employed to expose the context and characteristics of the included studies to facilitate comparison of similarities and differences across studies [18]. Further details of the review protocol have been published elsewhere [19]. The protocol was registered with PROSPERO (CRD42014007106).

We embedded an integrated knowledge translation (iKT) approach in our review, whereby we met with key knowledge users (e.g., ED clinicians, administrators, parents, and researchers) during each stage of the review to strengthen the relevancy of our research questions and tailor our recommendations. We employed a number of communication strategies to maximize engagement including email and face-to-face and teleconference meetings. Critical reflection of engagement was conducted through detailed documentation of team discussions at all meetings, outlining input from the different stakeholder groups, and underlying rationale for decisions made at each stage of the synthesis process. We also integrated a knowledge user check-in strategy, where at regular intervals during each meeting, we paused to summarize the discussion and solicit questions. Three key meetings included (1) a teleconference to refine the research question, (2) a face-to-face meeting to discuss the results of the preliminary analysis, and (3) a teleconference meeting to discuss findings and draft recommendations. Finally, authors from three of the included studies (all ED physicians from academic teaching hospitals) were contacted by email and invited to provide feedback on the preliminary findings. Expert feedback was provided through a brief telephone interview lasting 15–30 min.

We used the Behaviour Change Wheel (BCW) [20], a theory-based method for characterizing and designing behavior change interventions, as a guiding framework to examine the discharge communication interventions in the included studies. Pairs of two reviewers (KM, MB, AG, or JAC) independently coded the narrative descriptions of all interventions. A directed content analysis approach was used to classify intervention description according to the nine intervention function types (i.e., the mechanism by which the intervention is proposed to function) of the BCW [20]. Reviewers met to compare consistency after coding the first and third study and then after every five studies. Differences in coding were discussed to achieve consensus.

We also coded the 75 included studies to identify barriers and enablers to intervention implementation and effectiveness as reported by the study author. Full text articles were loaded into NVivo (QSR International) and were coded by two reviewers (JAC and AG) [21]. Reviewers met throughout the process to ensure consistent coding of barriers and enablers and to discuss sections that were challenging to code.

### Search strategy

The search strategy was developed in consultation with a library scientist and peer reviewed by a second library scientist from the Alberta Research Centre for Child Health Evidence (ARCHE; University of Alberta) prior to being implemented in three databases from their date of inception to July 7th, 2017: CINAHL, Medline, and Embase. Please see Additional file 1 for the list of all search strategy terms. We also hand-searched relevant emergency pediatric journals for articles published between January 1st, 2009 and October 31st, 2018: *Annals of Emergency Medicine, Academic Emergency Medicine*, *Pediatric Emergency Care*, *Journal of Emergency Medicine*, *and Journal of Emergency Nursing*. Additionally, a further search was conducted by emailing the ED administrators of the 15 Pediatric Emergency Research Canada (PERC) sites and the Chairs of the Pediatric Research in Emergency Departments International Collaborative (PREDICT), Pediatric Emergency Medicine Collaborative Research Committee (PEM-CRC), Pediatric Emergency Care Applied Research Network (PECARN), and the Paediatric Emergency Research in the United Kingdom & Ireland (PERUKI) and reviewing relevant national and international websites to identify existing discharge communication policies and procedures that might be relevant in a pediatric ED context.

### Study selection

Studies were included if they described or evaluated changes in the structure or process of care in the ED to enhance discharge communication as their primary objective. Quantitative, qualitative, and mixed methods study designs were eligible for inclusion. Studies were excluded if interventions took place outside of the ED or primary outcomes were not relevant to discharge communication. The published protocol paper for this review provides further details on the process of selecting studies [19]. Detailed inclusion and exclusion criteria for studies and abstracts can be found in Additional file 2.

### Data extraction

Data were extracted by teams of two independent reviewers using a customized data abstraction form and discrepancies were assessed by JAC. Full data extraction included study design details (i.e., year of publication, country, type of ED), detailed description of the intervention (i.e., description of individual components, reported use of theory or assumptions about causal mechanisms supporting the different components, duration, and timing of the intervention), implementation strategies (i.e., training or instructions provided to participants, timing), participant details (i.e., age of the child, illness presentation, parent characteristics, health care provider characteristics), and author reported factors and/or processes identified as impacting implementation. Our primary outcome of interest was any change in process, parent, healthcare provider, or patient outcome related to the discharge communication intervention. We extracted details about authored reported primary outcomes (i.e., timing, measures, target of the intervention). Data was managed using excel and intervention descriptions were exported to NVivo 10 (QSR International) for analysis [19].

### Quality assessment

Study quality was assessed at the study level by two independent reviewers using critical appraisal checklists from Joanna Briggs Institute (JBI) [22]. Each study was appraised using the appropriate checklist for the study design. JBI checklists are designed to assess the quality of a study’s methodologies and outcomes, and to identify potential sources of bias or confounding variables within the study [22]. Options for checklist responses included “yes,” “no,” “unclear,” or “not applicable.” Each reviewer used the JBI definitions and checklists and provided evidence from the article to support the reasoning for their scoring. At two points in the appraisal process, the reviewers met to meet consensus and resolve discrepancies. A third reviewer was consulted when discrepancies could not be resolved.

### Data synthesis

The primary aim of this review was to understand how and why discharge communication interventions and processes work in a pediatric ED context. To address this aim, we employed a number of data synthesis strategies proposed by Popay et al. [16] to examine the individual study findings. We developed summary tables, which outlined study design, clinical context, intervention components, intervention target, quality appraisal, outcome measures, and direction of effect. Findings of included studies did not lend themselves to quantitative analysis due to the wide range of intervention descriptions and outcome measures. We also developed structured textual summaries of all included studies that outlined primary objectives, details regarding context and setting, descriptions of any interventions and implementation strategies, relevant outcomes, and barriers reported by study authors. Content analysis was carried out on all extracted data to assist with identifying importing themes and gaps in the existing evidence.

## Results

**Fig. 1:**
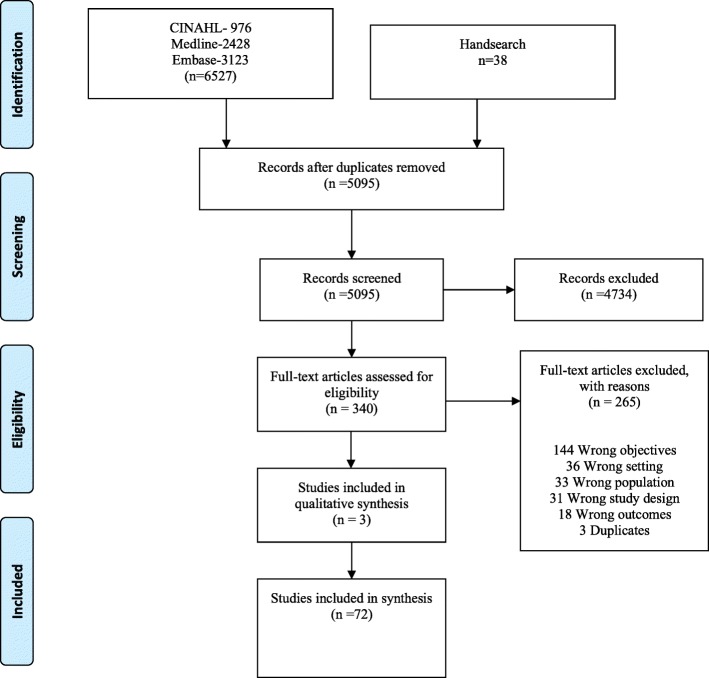
PRISMA diagram

A total of 5095 studies were identified by the search. Of these studies, 4734 studies were excluded at the title and abstract phase and 342 were eligible for full text screening. A total of 265 articles were excluded at the full text screening stage (see Additional File 3), resulting in 75 articles included in the review (Fig. 1). Half of the included papers were observational studies (*n* = 37) [23–59] and 47% (*n* = 35) were either randomized controlled trials (RCT) or quasi-experimental studies [60–94]. Three qualitative studies were also included [95–97]. Characteristics of the included studies are presented in Table 1. Detailed about excluded studies can be found in additional Table 2. Included studies covered a range of settings: pediatric ED, mixed ED (adult and pediatric), urban, rural, and academic teaching facilities. Although included studies focused on a variety of illness presentations, asthma (*n* = 20) was the most common [27, 29, 39, 42, 46, 61, 62, 65, 67, 70, 75, 77, 79–83, 85, 89, 90]. Other common illnesses were minor head injury (*n* = 12) [30, 34, 36, 44, 48, 50, 51, 56, 58, 68, 86, 94] and fever (*n* = 9) [35, 46, 64, 67, 68, 71, 72, 78, 88]. Outcomes varied across included studies but primarily involved improving parent adherence with discharge instructions or follow-up appointments (*n* = 12) [60, 61, 66, 75, 77, 81–85, 90, 93], improving parent knowledge of illness treatment (*n* = 7) [63, 65, 67, 68, 70–72], and reducing unnecessary return ED visits (*n* = 5) [62, 64, 69, 88, 89]. All studies were published from 1979 to 2018, with just over half (*n* = 41) of the studies published since 2009 [23–25, 30, 32–34, 37, 40, 42, 44–52, 55, 56, 59, 61, 64–68, 71–73, 75, 76, 79, 81, 83, 85, 92, 94, 96, 97].

**Table 1:** Description of included studies (*n* = 75)

Author, publication year	Study objective related to discharge communication	Study design	Appraisal checklist used	Country	Sample characteristics	Key findings as related to study objectives
Al-Harthy et al. 2016 [24]	To determine the delivery and usefulness of discharge instructions received in the pediatric ED.	Cross-sectional	Analytical cross-sectional study	Saudi Arabia	173 parents who had a child discharged from a pediatric ED and attended a follow-up appointment.	55% of parents received verbal discharge communication and 35% did not receive any form of instruction. The 6% of parents who received both verbal and written instructions were more knowledgeable about medication details compared the other groups.
Ali et al. 2012 [25]	To determine if discharge communication or poor provider-patient relationship were reasons for return visits to the ED within 72 h of initial visit.	Cross-sectional	Analytical cross-sectional study	USA	246 parents who returned to the ED within 72 h of the initial visit with their child (surveyed: *n* = 124, not surveyed: *n* = 122).	75% of parents returned because their child’s symptoms had not improved. Almost all parents (94%) thought their discharge communication were informative and were satisfied with their care on the initial visit.
Chacon et al. 1994 [26]	To assess parents’ reading level, assess written information resources provided in pediatric ED to ensure information was an appropriate reading level, and update resources based on the findings.	Cross-sectional	Analytical cross-sectional study	Canada	1022 parents who attended a pediatric ED with their child.	Half of the written discharge communications provided in the ED were written at a university level, much higher than the recommended grade 6–7 reading level. Parents found the updated written instructions provided useful information and were easy to read and understand.
Demorest et al. 2004 [31]	To assess parents’ memory of receiving poison prevention information during the ED visit.	Cross-sectional	Analytical cross-sectional study	USA	75 parents of children (< 6 years) who were brought to ED for acute accidental poisoning.	73% of parents did not recall receiving any information on poison prevention during their ED visit.
Grover et al. 1994 [57]	To examine parent recall of information and instruction provided during an ED visit.	Cross-sectional	Analytical cross-sectional study	USA	152 parents with children who presented to the ED with non-traumatic conditions.	25% of parents could not properly recall their child’s diagnosis. When one medication was prescribed, only 30% of parents could recall the name of the medication, and only 13% could properly name all medications when multiple were prescribed.
Hanson et al. 2017 [33]	To determine if an illustrated education sheet was a feasible method to convey information to parents and children in the ED.	Cross-sectional	Analytical cross-sectional study	USA	50 parents and their child (4–18 years) who presented to the pediatric ED.	Comic module was feasible to use in the ED and could be independently read in less than 11 min. Children < 12 years needed help. At 3-day follow-up, 86% of parents remembered all components taught in the comic educational module.
Johnson 1999 [36]	To determine if parents valued and utilized written health information provided during an ED visit.	Cross-sectional	Analytical cross-sectional study	Australia	200 parents of children who received pamphlets when their child presented at the ED for gastroenteritis, croup, application of bandage, or head injury.	95% of parents found the written information useful and 71% kept it for reference. Only half of parents indicated that a clinician reviewed the pamphlet with them during the ED visit.
Kaestli et al. 2015 [59]	To determine if the provision of customized drug information leaflets would improve parent knowledge.	Cross-sectional	Analytical cross-sectional study	France	125 French-speaking parents of children (0–16 years) who were discharged from ED (control: *n* = 56, intervention: *n* = 69).	Parents who received the drug information leaflets had significantly improved knowledge scores than controls about drug dosing (89.1% vs. 62.3%), frequency (85.5% vs. 57.9%), duration (66.7% vs. 34.2%), and indication (94.9% vs. 70.2%) (*p* ≤ 0.0001).
Lawson et al. 2011 [38]	To determine parents’ knowledge about child car seats and how many parents received car seat-specific discharge instructions in the ED.	Cross-sectional	Analytical cross-sectional study	USA	92 parents who attended the ED with their child following a car accident.	Most parents reported regular use of car seats for their child and knew of the requirements for car seats for children < 8 years. Only 18% of parents received information regarding child car seat safety during their ED visit.
Macy et al. 2013 [40]	To examine physicians’ awareness of and referrals to child passenger safety resources.	Cross-sectional	Analytical cross-sectional study	USA	638 ED physicians.	Physicians working in pediatric ED were significantly more likely to know about hospital resources to assist with child safety compared to physicians in non-pediatric centres (*p* < 0.001). More than half of physicians identified that they likely would not provide written discharge communication about car seats following a motor vehicle collision.
Melzer-Lange et al. 1998 [41]	To compare rates of follow-up care between ED violence related injuries and other presentations.	Cross-sectional	Analytical cross-sectional study	USA	426 medical records of adolescent patients (13–18 years) who presented to the pediatric ED.	Adolescents who presented with violence related injuries were less likely to receive discharge information about follow-up care compared to other ED presentations.
Philips 2009 [42]	To evaluate the implementation of a best practice-based asthma management policy.	Cross-sectional	Analytical cross-sectional study	Australia	100 parents who presented to the ED with a child (< 17 years) with asthma.	Parents’ asthma management greatly improved following the intervention. Responses to the follow-up survey improved after the intervention implementation.
Ray et al. 2016 [45]	To determine how adolescents who attend the ED prefer to receive discharge and follow-up communication.	Cross-sectional	Analytical cross-sectional study	USA	439 adolescents (14–19 years) who attended the pediatric ED.	78% of adolescents preferred receiving ED discharge communication and follow-up appointment information via electronic means, with 57% of these participants preferring to receive this information in both printed and electronic formats.
Sargant and Milsom 2012 [47]	To develop a cellphone “app” to provide additional written and oral education for common ED presentations.	Cross-sectional	Analytical cross-sectional study	UK	520 parents who waited for care for at least 30 min in the ambulatory zone between 10 am and 10 pm.	97% of survey respondents thought phone apps would be useful in providing additional information following an ED visit.
Stevens et al. 2010 [50]	To identify if parents were able to recognize post-concussive symptoms following ED discharge.	Cross-sectional	Analytical cross-sectional study	USA	105 parents of children (5–17 years) who were treated at pediatric ED for concussion.	Almost 70% of parents were not able to identify post-concussive symptoms in their child and 46.6% did not attribute their child’s symptom(s) to their concussion.
Wahl et al. 2011 [52]	To identify parents’ thoughts on the current methods of ED discharge communication and examine families’ needs for health information.	Cross-sectional	Analytical cross-sectional study	UK	1046 parents of children presenting to the pediatric ED.	80.5% of parents enjoyed the current written discharge communication, but 47.9% noted a video format would be useful. Over half of parents also used friends and family (59.5%) and the internet (57.5%) for additional information following ED discharge.
Waisman et al. 2003 [53]	To examine parents’ level of understanding of discharge communication and factors that influence their understanding.	Cross-sectional	Analytical cross-sectional study	Israel	287 parents of children discharged home from the ED.	75% of parents understood their child’s diagnosis and 84.5% understood the treatment plan. The use of medical terms and jargon was the primary reason for not fully understanding the information.
Waisman et al. 2005 [54]	To examine the impact of diagnosis-specific discharge communication sheets on parents’ understanding of ED discharge communication.	Cross-sectional	Analytical cross-sectional study	Israel	95 parents of children discharged home from the ED.	Parents were significantly more likely to fully understand treatment instructions (*p* = 0.025) with the use of the information sheets, but it did not significantly improve understanding of their child’s diagnosis (*p* = 0.54).
Zonfrillo et al. 2011 [55]	To examine ED physicians’ knowledge of child passenger safety recommendations and their provision of this information.	Cross-sectional survey and retrospective chart review	Analytical cross-sectional study	USA	317 physicians who reported routinely treating children involved in motor vehicle collisions.	85% of physicians thought it was important to include discharge communication about child passenger safety recommendations, although a review of medical charts showed only 8.6% of visits contained child passenger information.
Fung et al. 2009 [58]	To review the content of written discharge information provided to mild traumatic brain injury patients and their caregivers, compared to six key evidence-based recommendations.	Cross-sectional study	Analytical cross-sectional study	Canada and USA	10 ED in New York and five ED in Ontario	All of the written forms included in the study varied widely in the content and delivery of discharge information. Only one form included all six recommendations. The average reading level of the resources was 8.2, compared to the ideal level of 5–6.
Hawley et al. 2012 [34]	To evaluate printed resources provided to parents of children with head injuries.	Cross-sectional study	Analytical cross-sectional study	UK	211 hospitals provided copies of their printed head injury resources for the study.	The quality of information provided in the printed resources varied. Thirty-three resources received a perfect score and an additional 33 resources were considered to include questionable information.
Qureshi et al. 2013 [44]	To evaluate written discharge information provided to parents of children with head injuries with the recommendations from the Scottish Intercollegiate Guideline Network (SIGN).	Cross-sectional study	Analytical cross-sectional study	Scotland	36 hospitals provided copies of the written head injury information.	The quality of written information for head injuries varied widely, with anywhere from 4 to 20 of the 20 SIGN urgent symptoms to monitor being listed (median score of 10/20 guidelines identified). Two leaflets contained the majority of head injury guidelines and four pamphlets provided erroneous or unclear details.
Thomas et al. 2017 [51]	To determine the effect of reinforcing written discharge communication with verbal instruction on parents’ recall of discharge communication.	Nested observational study	Analytical cross-sectional study	USA	93 parents of pediatric patients presenting to the ED with mild traumatic brain injury.	Receiving verbal instructions in addition to written handouts on post-concussion care was associated with improved recall of instructions (67% vs 44%; *p* < 0.05).
Cheng et al. 2002 [27]	To assess factors associated with metered-dose inhaler spacers (MDIS) use at home in children with asthma who received asthma education during their ED visit.	Prospective observational	Analytical cross-sectional study	Australia	73 parents of children (12 months–16 years) who attended a pediatric ED with asthma.	Compliance with MDIS use at home was significantly higher when MDIS was used during the ED visit (*p* = 0.05), the parents received written instructions on how to use MDIS at home (*p* = 0.003), and receiving a smaller sized spacer (*p* ≤ 0.001). Parent preferences and size of the spacers were factors in non-compliance at home.
Crain et al. 1999 [29]	To assess the development and use of a tool to code history taking and discharge planning during asthma related ED visits.	Prospective observational	Analytical cross-sectional study	USA	154 parents of children (4–9 years) who presented to an ED with asthma.	Half of the items (7/13) on the ED asthma care checklist were endorsed by over 90% of physicians and allergists. Very few elements from the checklist were identified during ED visits.
Hemphill et al. 1998 [35]	To identify factors associated with compliance with recommended follow-up after ED visits.	Prospective observational	Analytical cross-sectional study	USA	330 parents of children (3 months–10 years) who presented to the ED with fever.	Parents who received an appointment time were 2.5 times as likely to comply with follow-up than parents who had to make an appointment with a pediatrician.
Pizarro et al. 1979 [43]	To reduce hospitalizations by evaluating the effectiveness of training mothers in the ED to provide oral rehydration at home for their ill infants.	Prospective observational	Analytical cross-sectional study	Costa Rica	100 infants (18 days–20 months) who presented to the ED with diarrhea.	Providing oral rehydration solutions and including parents in the rehydration care at the ED was beneficial in 92% of cases, and helped reduce length of hospital stay.
Samuels-Kalow et al. 2013 [46]	To examine how language impacted discharge communication and parents’ understanding of the information.	Prospective observational	Analytical cross-sectional study	USA	210 parents of children (2 months–2 years) presenting at a pediatric ED with fever and/or respiratory illness.	32% of parents demonstrated dosing errors during discharge, despite receiving written instructions for dosing. Spanish- vs English-speaking parents were significantly more likely to make a dosing error (54% vs. 25%).
Sauer et al. 2012 [49]	To determine if providing detailed information and a telephone hotline to help schedule follow-up appointments could reduce return ED visits.	Cohort study	Cohort	USA	2120 patients attending a pediatric ED.	Patients who received the intervention were less likely to return to the ED following discharge. Only 10% of patients used the telephone hotline to schedule follow-up appointments.
Akinsola et al. 2017 [23]	To determine if a quality improvement project improved written discharge communication.	Retrospective cohort study	Cohort	USA	1763 charts of children who presented to the ED with an emergency index score of 2 or 3.	The intervention significantly improved each of the eight individual (91–99% improvement) written discharge communication elements. There was a significant improvement in providing all eight elements of discharge communication to patients (*p* < 0.001).
Chorley 2005 [28]	To determine the ED discharge information provided for ankle sprains, follow-up appointments, and differences between instructions provided to adults and pediatric patients.	Retrospective cohort study	Cohort	USA	374 patient charts.	None of the patients received all of the discharge communication components for ankle sprains. Younger patients were more likely than adult patients to receive the information to rest, ice, compress, elevate, and use analgesia medications.
De Maio et al. 2014 [30]	To assess the discharge information provided to pediatric patients following an ED visit for concussion.	Retrospective cohort study	Cohort	USA	218 patients (6–18 years) who had presented to a pediatric ED with a possible concussion.	Most patients were not told to restrict activities following their concussion, although activity restriction was more likely to be discussed with patients with sports-based injuries.
Gaucher et al. 2011 [32]	To examine the effect of nurse counseling on common childhood illnesses on the return rates of patients who leave the ED without being seen by a physician.	Retrospective cohort study	Cohort	Canada	10,323 children (< 19 years) who were triaged by a nurse but left the ED before being seen by a physician.	Nurse counseling for patients leaving the ED prior to seeing a physician significantly reduced likelihood of return to the ED within 48 h than those who were not counseled by a nurse (8.1% vs. 6.1%; OR 0.78, 95% CI 0.65–0.94).
Lawrence et al. 2009 [37]	To determine if standardized typed diagnosis-specific instructions decreased return visits to the pediatric ED.	Retrospective cohort study	Cohort	USA	979 children who presented to the ED.	Diagnosis-specific typed discharge communication did not reduce inappropriate return (3% vs. 2.3%) or medically unnecessary (15% vs. 13%) visits to the ED compared to handwritten instructions.
Ly and Dennehy 2007 [39]	To examine ED management of pediatric asthma and if it followed national guidelines.	Retrospective cohort study	Cohort	USA	141 patients (1–17 years) who presented to the ED with asthma.	Guidelines for discharge communication were not followed consistently. About two thirds (67.4%) did not receive asthma device training, 80.1% did not receive a written action plan, and 50% of patients over the age of six did not receive a peak flow meter.
Sarsfield et al. 2013 [48]	To determine if follow-up care and activity limitations were included in discharge information provided for head injuries.	Retrospective cohort study	Cohort	USA	204 children (2–18 years) who presented to the trauma center with minor head injuries, traumas, or concussions.	95% of patients were informed about follow-up care and 82% received information about anticipatory guidance.
Upchurch et al. 2015 [56]	To determine if a concussion-based intervention improved discharge communication provided in the ED.	Retrospective cohort study	Cohort	USA	497 children who presented to the pediatric ED between 2004 and 2012 with a sports-related concussion.	66% of patients received appropriate discharge communication, which improved to 75% after 2010 (*p* = 0.179). Referrals to specialists significantly improved after 2010 (*p* < 0.001).
Samuels-Kalow et al. 2015 [97]	To determine the impact of “teach-back” in the ED based on health literacy level.	Interviews	Qualitative	USA	31 interviews with parents of pediatric asthma patients in the ED (15 with limited literacy); 20 interviews with adult ED asthma patients (12 with limited literacy).	Across all participants and health literacy levels, asthma teach-back in the ED was viewed as an important way to confirm understanding of information, helped to reinforce key messages, and improved communication with health care providers.
Samuels-Kalow et al. 2016 [96]	To identify barriers and facilitators of the ED discharge communication process.	Interviews	Qualitative	USA	31 interviews with parents of pediatric asthma patients in the ED (15 with limited literacy); 20 interviews with adult ED asthma patients (12 with limited literacy).	Participants appreciated simple visuals and language, consistency in terms used, and having dedicated time for discharge communication during the ED visit. Some parents of pediatric patients also discussed feeling insecure disclosing to health care providers when they did not fully understand the content of discharge communication.
Leickly et al. 1998 [95]	To examine factors that affect adherence to asthma treatment plans.	Interviews and chart review	Qualitative	USA	344 English- and Spanish-speaking children (4–9 years) presenting at inner city ED with asthma.	Interviews indicated that parents might overuse the ED due to lack of planning for acute asthma symptoms. Asthma management follow-up was also influenced by PCP recommendation of scheduling an appointment.
Boychuk et al. 2006 [70]	To develop and implement an ED-based, asthma education program based on National Asthma Education and Prevention Program (NAEEP) guidelines.	Quasi-experimental	Quasi-experimental studies (non-randomized experimental studies)	USA	Phase 1: 590 patients (< 18 years) Phase 2: 313 patients (< 18 years)	Asthma education can be provided effectively in ED. The intervention was also effective at connecting ED and community physicians to encourage asthma education and treatment with patients.
Considine and Brennan 2007 [78]	To examine the effect of a staff educational intervention on discharge advice provided to parents leaving the ED.	Quasi-experimental	Quasi-experimental studies (non-randomized experimental studies)	Australia	40 parents of children who presented to the ED with fever.	Structured education of ED nurses through two 30-min tutorials improved discharge advice given to parents.
Kruesi et al. 1999 [87]	To examine if injury prevention education could reduce access to lethal means.	Quasi-experimental	Quasi-experimental studies (non-randomized experimental studies)	USA	103 parents who brought a child (6–19 years) to the ED to receive a mental health assessment (control: *n* = 62, intervention: *n* = 41).	The intervention group was significantly more likely to take action to limit access to prescription drugs, over-the-counter medications, and firearms (*p* ≤ 0.05).
O’Neill-Murphy et al. 2001 [88]	To examine if a fever education intervention could help reduce parent anxiety and improve home management of fevers.	Quasi-experimental	Quasi-experimental studies (non-randomized experimental studies)	USA	87 parents of children (3 months–5 years) who presented to the ED with a fever (control: *n* = 43, intervention: *n* = 44).	Both groups had reduced anxiety levels at post-test. There were no significant differences in knowledge scores between groups.
Patel et al. 2009 [76]	To improve parent recall of verbal discharge information with the use of a bilingual discharge facilitator.	Quasi-experimental	Quasi-experimental studies (non-randomized experimental studies)	USA	325 English- or Spanish-speaking parents who brought a child (3 months–18 years) to the ED (control: *n* = 270, intervention: *n* = 192).	Parents in the discharge facilitator group could recall more signs and symptoms than standard discharge group (mean 4.3 vs 3.3; mean difference 1.0)
Petersen et al. 1999 [77]	To determine if an asthma education tool improved rates of follow-up care.	Quasi-experimental	Quasi-experimental studies (non-randomized experimental studies)	USA	Children (5–16 years) who presented to the ED with asthma (before: *n* = 114; after: *n* = 97).	There was a significant increase in proportion of children told to return for follow-up (54% to 81%, p = < 0.0001), but there was no difference in follow-up rates (7% to 6%) following implementation of the tool.
Porter et al. 2006 [80]	To determine the effectiveness of an asthma information kiosk on parent satisfaction, and clinician use of the kiosk information.	Quasi-experimental	Quasi-experimental studies (non-randomized experimental studies)	USA	273 parents were included in the implementation phase study (control: *n* = 131, intervention: *n* = 142).	Parents’ satisfaction with health care providers did not increase with the use of the kiosk (0.8 vs. 0.7). When clinicians used the kiosk information, there were fewer reported issues (0.6 vs. 1.1).
Rotheram-Borus et al. 2000 [91]	To implement a specialized ED and outpatient suicide prevention intervention to decrease suicidal behaviors.	Quasi-experimental	Quasi-experimental studies (non-randomized experimental studies)	USA	140 females (12–18 years) who had attempted suicide and their parent presented in the ED (control: *n* = 75, intervention: *n* = 65).	Rates of reattempts (*n* = 11 control, *n* = 6 intervention) and suicide ideation across both groups were consistently low during the 18 months follow-up.
To et al. 2010 [79]	To determine if an asthma information card would improve asthma management in the ED.	Quasi-experimental	Quasi-experimental studies (non-randomized experimental studies)	Canada	432 children (2–17 years) who presented to the ED with an acute asthma exacerbation (control: *n* = 278, intervention: *n* = 154).	Use of an asthma management reminder card improved the provision of oxygen (*p* = 0.00074), salbutamol (*p* ≤ 0.0001), ipratropium bromide (*p* = 0.0161), and oral corticosteroids (*p* ≤ 0.0001) in the ED. However, there were no significant differences in providing information about asthma (*p* = 0.5940) or follow-up visits (*p* = 0.0705) between groups.
Williams et al. 2013 [75]	To improve parents’ understanding of their child’s asthma severity during the ED visit.	Quasi-experimental	Quasi-experimental studies (non-randomized experimental studies)	USA	Children with asthma aged 1–17 years (controls: *n* = 216, intervention: *n* = 70).	Parents who received the asthma education intervention were more likely to attend follow-up care in the week following discharge (*p* ≤ 0.0001).
Wood et al. 2017 [71]	To determine if providing video discharge communication would improve parents’ knowledge.	Quasi-experimental	Quasi-experimental studies (non-randomized experimental studies)	USA	83 parents of children (up to 21 years) who presented to the ED with gastroenteritis, fever, or bronchiolitis (control: *n* = 42, intervention: *n* = 41).	Across the three illness presentations, parents who received the intervention had significantly higher knowledge scores compared to parents who received standard discharge communication (gastroenteritis 73% vs. 57%, *p* = 0.005; fever 76% vs. 69%, *p* ≤ 0.001; bronchiolitis 64% vs. 49%, *p* = 0.025).
Gorelick et al. 2006 [62]	To compare low, medium, and high intensity asthma interventions on ED return visits and quality of life (QOL).	RCT	RCT	USA	352 children who presented to the ED with acute asthma (control: *n* = 116, intervention group 1: *n* = 118, intervention group 2: *n* = 118).	No differences between study groups for return visits to the ED (17.3% vs. 17.9% vs. 19.2%, *p* = 0.94).
Stevens et al. 2012 [66]	To determine the effect of a childhood pain management video on parents’ knowledge and pain meds use following ED discharge.	RCT	RCT	USA	100 parents of children (1–18 years) who presented to the ED with pain (control: *n* = 41, intervention: *n* = 59).	The intervention group had significantly improved knowledge of pain (*p* = 0.04), the impact of pain on function (*p* ≤ 0.01), and misconceptions around pain medications (*p* ≤ 0.01).
Baker et al. 2009 [64]	To determine if an educational video about home management of fevers could reduce return ED visits.	Randomized cohort study	RCT	USA	280 parents who presented to the ED with a child (3–36 months) with a fever (control: *n* = 140, intervention: *n* = 140).	Intervention group had significantly improved responses to appropriate knowledge and treatment of fever (*p* ≤ 0.0001). No difference between groups for return ED visits (*p* = 0.46).
Zorc et al. 2003 [82]	To determine if scheduling follow-up appointments during the ED visit improves follow-up rates.	Randomized trial	RCT	USA	286 parents with children (2–18 years) who were treated for asthma and discharged from the ED. Eight potential participants were excluded; leaving 139 participants included in each the control and the intervention group.	The intervention group was more likely to follow-up with a primary health care provider (64% vs. 46%, *p* = 0.003), and in a shorter time frame (13 vs. 54 days, *p* = 0.004).
Asarnow et al. 2011 [92]	To evaluate a suicide prevention intervention to improve use of outpatient care and decrease suicide attempts.	RCT	RCT	USA	181 suicidal youths (10–18 years) (control: *n* = 92, intervention: *n* = 89).	Intervention group significantly more likely to attend outpatient care (92% vs. 76%, *p* = 0.004), but was not significantly associated with decreased suicide attempts (13% vs. 13%).
Bloch and Bloch 2013 [67]	To determine if a video providing discharge information improved parents’ knowledge of the ED visit and follow-up care.	RCT	RCT	USA	436 parents who presented to the ED with a child (< 18 years old) with asthma, fever, or vomiting/diarrhea (control: *n* = 220, intervention: *n* = 216).	Intervention group had significantly higher knowledge scores at (12.2 vs. 8.9, *p* < 0.0001) and following (11.1 vs. 7.9, *p* ≤ 0.0001) ED visit.
Brooks et al. 2017 [90]	To determine how useful symptom-specific discharge instructions were to parents in managing their child’s concussion symptoms and activities.	RCT	RCT	USA	114 parents of children (7–17 years) who presented to the ED with a concussion (control: *n* = 56, intervention: *n* = 58).	The intervention group found the discharge instructions significantly more helpful compared to the control group (*p* < 0.05). Although the control group were more likely to contact their PCP regarding concussion follow-up care (*p* < 0.05).
Chande et al. 1996 [69]	To determine if educating parents could reduce unnecessary ED visits.	RCT	RCT	USA	130 parents who presented to the ED with a child with a minor illness (control: *n* = 61, intervention: *n* = 48).	Six months following the initial ED discharge, there was no significant difference between groups returning to the ED (30% vs. 26%, *p* = 0.68).
Cushman et al. 1991 [86]	To encourage the purchase of helmets following a visit to the ED with a child with a bike injury.	RCT	RCT	Canada	334 parents who presented to the ED with a child who had a bike injury (control: *n* = 173, intervention: *n* = 161).	There was no significant difference in helmet purchases between groups (9.3 vs. 8.1, *p* = 0.69).
Delp and Jones, 1996 [60]	To determine if parent comprehension was improved with the use of cartoon illustrations with discharge information.	RCT	RCT	USA	234 patients and parents (of children under 14 years) who were released from the ED and had a telephone (control: *n* = 129, intervention: *n* = 105).	The intervention group was more likely to have read the discharge information (98% vs 79%, *p* ≤ 0.001), properly answer questions about care (46% vs 6%, *p* ≤ 0.001) and adhere to discharge information (77% vs. 54%, *p* ≤ 0.01).
Ducharme et al. 2011 [61]	To determine if providing written action plan and a prescription improved adherence to asthma controller medication.	RCT	RCT	Canada	219 children diagnosed with asthma who had received albuterol nebulization treatment at least once, and were discharged home with albuterol and fluticasone in metered-dose inhalers (control: *n* = 110, intervention: *n* = 109).	Compliance with asthma discharge information was comparable between groups at 14 days, but by 4 weeks post-ED visit, the intervention group had double the compliance rate of the control group (mean group difference 16.1%).
Hart et al. 2015 [72]	To determine if an interactive online education resource improved parents’ knowledge of fever.	RCT	RCT	Canada	203 parents of children who presented to the ED with fever (control: *n* = 67, interactive website intervention: *n* = 66, standard website intervention = 70).	Parents who received either intervention had significant improvements in mean knowledge scores compared to control group (3.9 vs. 3.5 vs. 0.2, *p* ≤ 0.001). No differences in knowledge scores between the two intervention groups (*p* = 0.55).
Hussain-Rizvi et al. 2009 [81]	To determine if parents providing MDIS with physician supervision improved compliance with asthma home management.	RCT	RCT	USA	86 children (1–5 years) who had presented to the ED with acute asthma exacerbation (control: *n* = 46, intervention: *n* = 40).	Outcomes were comparable between groups. However, the intervention group had better MDIS use following ED discharge (95.0% vs. 71.7%, *p* = 0.04).
Isaacman et al. 1992 [74]	To determine if offering verbal or verbal and written discharge information would improve information recall.	RCT	RCT	USA	197 parents of a child with otitis media (control/usual care: *n* = 84, standardized verbal: *n* = 52, standardized verbal + written intervention: *n* = 61).	Parents in all groups improved recall of medication data, signs of improvement, and worrisome signs from exit interview to day 1 and 3. Standardized instruction groups performed significantly better than control in all areas except medication data at all time points. Verbal + written group significantly better than written alone on signs of improvement at exit interview only (56.9% vs. 25.3%; *p* < 0.05)
Ismail et al. 2016 [68]	To determine if providing video discharge information would improve recall and comprehension.	RCT	RCT	USA	63 parents who presented to the ED with a child with either a fever or closed head injury (control: *n* = 32, intervention: *n* = 31).	Intervention group had higher post-test scores compared to control group (88.89 vs. 75.73, *p* ≤ 0.0001).
Jones et al. 1989 [84]	To increase compliance with follow-up care through the use of health belief model (HBM) clinical nursing interventions.	RCT	RCT	USA	59 patients who presented to the ED with otitis media (control: *n* = 19, HBM phone intervention: n = 12, HBM clinical intervention: *n* = 14, HBM phone and clinical intervention: *n* = 14).	All intervention groups had higher rates of scheduling (90% vs 26%, *p* ≤ 0.001) and keeping (73% vs. 21%, *p* ≤ 0.001) a follow-up appointment compared to the control group. No significant differences in compliance rates between the three intervention groups.
Komoroski et al. 1996 [93]	To determine the effectiveness of two interventions provided prior to ED discharge aimed at improving rates of follow-up care.	RCT	RCT	USA	253 patients or parents of pediatric patients who presented to the ED with nonurgent conditions which required follow-up (control: *n* = 89, simple intervention: *n* = 85, intense intervention: *n* = 79)	Participants in the intense intervention group had the highest follow-up rates. The simple intervention group (*p* < 0.001) and the intense intervention group (*p* < 0.001) both had significantly higher rates compared to the control group. Those in the intense intervention group also had higher follow-up rates compared to the simple intervention, but it was not statistically significant.
LeMay et al. 2010 [73]	To test an education intervention about pediatric pain 24 h following ED discharge.	RCT	RCT	Canada	195 parents who visited the ED with a child with a musculoskeletal injury, burn, deep laceration, or acute abdominal pain (control: *n* = 97, intervention: *n* = 98).	No differences between groups for pain intensity at triage (5.4 vs. 5.11) and 24 h post-discharge (3.3 vs. 3.1). No differences in the level of unpleasantness experienced at triage (4.9 vs. 4.8) or 24 h post triage (3.0 vs. 3.1) (*p* = 0.05 for all tests).
Macy et al. 2011 [65]	To determine if a discharge communication video about asthma improved parents’ knowledge.	RCT	RCT	USA	129 parents of a child (2–14 years) who presented to the ED with asthma (control: *n* = 67, intervention: *n* = 62).	Parents with low literacy had improved asthma knowledge in both the intervention and control groups (*p* ≤ 0.0001). Parents with adequate health literacy had improved asthma knowledge in the intervention group, but this was not significant.
Scarfi et al. 2009 [83]	To determine if providing an allergen skin test for asthma and its results during an ED visit would improve follow-up rates at an asthma clinic.	RCT	RCT	USA	77 children (2–12 years) who presented to the ED with an acute asthma exacerbation (control: *n* = 39, intervention: *n* = 38).	The intervention group was 2.6 times more likely to schedule and maintain their follow-up appointments at an asthma clinic.
Smith et al. 2006 [90]	To determine if providing asthma coaching and incentives would improve rates of follow-up care with a PCP.	RCT	RCT	USA	92 parents who presented to the ED with a child with an acute asthma exacerbation (control: *n* = 42, intervention: *n* = 50).	Following ED discharge, there were no differences in follow-up rates between intervention and control groups (22% vs. 23.8%, *p* = 0.99).
Sockrider et al. 2006 [89]	To test a tailored asthma management intervention to improve parent confidence and reduce return ED visits.	RCT	RCT	USA	464 parents with children (1–18 years) who presented to the ED with an acute asthma exacerbation (control: *n* = 201, intervention: *n* = 434).	Following ED discharge, the intervention group had higher confidence in preventing their child’s asthma symptoms (*p* = 0.05) and preventing symptoms from getting worse (*p* = 0.03). The intervention group also had significantly lower rates of return ED visits for intermittent asthma (odds ratio = 0.32, 95% CI 0.12–0.88).
Yin et al. 2008 [63]	To determine if the use of illustrated health literacy interventions helped decrease liquid medication dosing errors.	RCT	RCT	USA	245 children (30 days–8 years) and parents who were given a prescription for a liquid medication in the pediatric ED (control: *n* = 121, intervention: *n* = 124).	The intervention group had fewer daily dosing errors (0% vs. 15.1%, *p* = 0.007) and higher use of standardized dosing instrument for daily (93.5% vs. 71.7%, *p* = 0.008) and as-needed (93.7% vs.74.7%, *p* = 0.002) dosing.
Zorc et al. 2009 [85]	To develop and test a 3-part asthma intervention to improve rates of follow-up care.	RCT	RCT	USA	439 parents with an asthmatic child (1–18 years). Six participants were excluded, leaving 216 participants in the control group and 217 participants in the intervention group.	The intervention group was more likely to support and believe in the benefits of seeking follow-up care with a PCP, but there were no significant differences in follow-up care rates between groups.

*CI* confidence interval, *ED* emergency department, *MDIS* metered-dose inhaler spacers, *RCT* randomized controlled trial, *PCP* primary care provider

**Table 2:** Description of intervention studies (*n* = 44)

Author, publication year	Intervention type	Description of intervention	Intervention target	Who delivered intervention	Description of primary outcome	Direction of effect
1 Intervention function						
Baker et al. 2009 [64]	Education	Parents watched a video (11 min) about how to manage child’s fever at home.	Parent	Research team	Reducing return ED visits.	+
Bloch and Bloch 2013 [67]	Education	Parents watched a video (3 min) regarding their child’s illness in addition to standard discharge communication. Parents were provided the opportunity to ask clarifying questions to a clinician prior to leaving the ED.	Parent	Medical student volunteers	Parent comprehension of treatment plan and follow-up information.	+
Boychuk et al. 2006 [70]	Education	Parents watched a video (6 min) outlining asthma and asthma management techniques, in addition to conversations with ED staff about the importance of asthma management and treatment, a review or written or verbal instructions, and a demonstration of how asthma affects the lungs. Health care providers also received asthma education.	Patient, parent, and health care provider	Research team, and physicians	Improving asthma management through written action plans and medications.	+
Brooks et al. 2017 [90]	Education	Parents received written discharge instructions specific to their child’s concussion level and symptoms they experienced.	Parent	Research team	Use of discharge instructions.	+
Chande et al. 1996 [69]	Education	Parents watched a video (10 min) and received an information booklet on pediatric illnesses. A research assistant reviewed the booklet with parents and answered parent questions.	Parent	Research team	Reducing return ED visits.	–
Cheng et al. 2002 [22]	Education	Caregivers received education about asthma and a demonstration of an appropriate MDIS technique during the ED visit.	Parent	Discharge facilitator and ED clinicians	Improving rates of MDIS use.	+
Considine and Brennan 2007 [78]	Education	Nurses in the ED attended two tutorials that reviewed the physiology and treatment of fevers to improve the content of discharge communication provided to parents.	Health care provider	Online tutorial	Information caregivers received about fever as part of their discharge instructions.	+
Delp and Jones 1996 [60]	Education	Parents received cartoon wound care instructions in addition to standard written instructions.	Patient and parent	Physicians	Patient compliance with ED discharge instructions.	+
Hanson et al. 2017 [29]	Education	Patients and parents received cartoon pain management information.	Patient and parent	Research team	Parent recall of information provided in the cartoon.	+
Hart et al. 2015 [72]	Education	Parents received one of two interventions. One intervention group received access to a standard fever education website. The second intervention group received accessed to an interactive fever education website.	Parent	Research team	Parent knowledge of fever.	+ for both interventions
Hussain-Rizvi et al. 2009 [81]	Modeling	Physician demonstrated for the parent how to use of the albuterol MDIS. The parent then provided the treatment while under observation.	Parent	Physicians	Adherence to MDIS use at home following ED discharge.	+
Gaucher et al. 2011 [28]	Education	Parents who notified the triage nurse they were leaving without being seen by a physician received information about their child’s illness and when to seek additional care and return to ED	Parent	Triage nurse	Rates of return ED visits within 48 h of the initial visit.	+
Isaacman et al. 1992 [74]	Education	Parents whose child presented with otitis media received one of two interventions. One intervention group received verbal instructions. The second intervention group received the same verbal instructions in addition to a written copy of discharge communication.	Parent	Residents and medical students	Parent recall of medications and signs to monitor at home.	+ for both interventions
Ismail et al. 2016 [68]	Education	Parents watched a video (3–5 min) about fever or closed head injury in addition to standard verbal discharge communication. Parents were also given the opportunity to ask questions to clinicians prior to leaving ED.	Parent	Clinicians	Parent comprehension of diagnosis and follow-up care.	+
Kaestli et al. 2015 [55]	Education	Parents received written information regarding drug dosing, indication, and frequency of administration.	Parent	Research team	Improving comprehension of prescribed drug usage.	+
LeMay et al. 2010 [73]	Education	Parents received a booklet about pain management and a bookmark printed with pain scale information in addition to standard care.	Parent	Research team	Children’s pain and parents’ perceptions of pain management 24 h following ED visit.	No change
Macy et al. 2011 [65]	Education	Parents watched a video (20 min) about an asthma management.	Parent	Health care providers and research assistants	Parent knowledge of asthma information provided during ED visit.	+
Patel et al. 2009 [76]	Education	Parents received verbal reinforcement of discharge communication from a discharge facilitator in addition to standard written information.	Parent	Discharge facilitator	Parent recall of discharge instructions.	+
Petersen et al. 1999 [77]	Education	Parents received personalized written asthma information about signs of asthma attack, medication doses, and following up with a PCP within 72 h of the ED visit.	Parent	Respiratory care providers and clinicians	Compliance with attending follow-up appointment and length of time before appointment was made.	No change
Porter et al. 2006 [80]	Environmental Restructuring	Parents used an interactive asthma kiosk to document asthma symptoms and care needs. Information generated from the kiosk was shared with ED clinicians through a reminder summary on chart.	Parent and health care provider	Research team and clinicians	Parent satisfaction with care and providers’ adoption of guideline-endorsed process measures.	No change
Stevens et al. 2012 [66]	Education	Parents watched a video (6 min) that provided information about common myths and misunderstandings surrounding home management of pain.	Parent	Research team	Parent use of pain management information provided at the ED following discharge.	+
Thomas et al. 2017 [47]	Education	Parents received verbal reinforcement of written discharge instructions.	Parent	Research team	Parent understanding of discharge instructions.	+
To et al. 2010 [79]	Environmental Restructuring	Health care providers received an evidence-based asthma guideline reminder card.	Health care provider	Research team	Changes to asthma medication, asthma education, and discharge planning provided to families in the ED.	+/ No change/ No change
Waisman et al. 2005 [50]	Education	Parents received a written information sheet regarding their child’s illness.	Parent	Physicians	Parent understanding of discharge instructions.	+
Williams et al. 2013 [75]	Education	Parents received an illustrated scale and were educated about their child’s asthma severity score along with standard discharge communication.	Parent	Research team and ED clinical providers	Parent compliance with scheduling follow-up care.	+
Wood et al. 2017 [71]	Education	Parents watched a video (3–5 min) specific to their child’s illness, in addition to standard verbal discharge communication and a written information sheet.	Parent	ED nurses from an evidence-based practice project	Parent comprehension of their child’s illness and treatment.	+
Zorc et al. 2003 [82]	Enablement	Researchers with the study brought parents to a phone in an attempt to get them to schedule follow-up care with their PCP.	Parent	Research team	Parent follow-up with PCP and improvements in asthma-related health outcomes and medication use.	+
2 Intervention functions						
Cushman et al. 1991 [86]	Education + Enablement	Physicians received a cue card with information to counsel parents on helmet use. Parents were provided with pamphlets and a card with the names and addresses of retailers selling helmets to take home to encourage the use and purchase of helmets. Follow-up phone calls were made to check if helmets had been purchased and to provide additional counseling, if needed.	Parent	Physicians	Measuring the purchase and use of bicycle helmets.	No change
Jones et al. 1989 [84]	Education + Enablement	Parents received one of three interventions. Group 1 received standard care in addition to a follow up telephone call. Group 2 received counseling during the ED visit with no follow-up call. Group 3 received both counseling in the ED and a follow-up call.	Parent	Research nurse and clinical nurse	Compliance with scheduling and attending a follow-up appointment based on a referral recommendation.	+
Kruesi et al. 1999 [87]	Education + Environmental Restructuring	Parents were informed of their child’s increased risk of suicide. Staff also educated and problem solved with parents to try and reduce suicide risk by limiting access to lethal means. Additionally, a safe disposal site was created for parents to encourage the removal of guns from the house.	Parent	ED staff	Reducing access to lethal means.	+
O’Neill-Murphy et al. 2001 [88]	Education + Modeling	Parents received a review of written fever information, had a discussion to have their questions answered, and received instructions and a demonstration of proper thermometer use.	Parent	Research team	Parent anxiety and rate of return ED visits for fever.	+
Pizarro et al. 1979 [39]	Education + Enablement	Following administration of oral rehydration fluid at the hospital, parents were sent home with the oral rehydration solution and were instructed of signs to monitor that would require a return visit to the ED.	Parent	ED nurses and interns	Reducing hospital length of stay.	+
Philips 2009 [38]	Education + Environmental Restructuring	Parents received asthma education packages, asthma discharge plans, and were provided with a spacer if their child did not have one.	Parent	ED staff	Improving parent’s treatment and home management of asthma.	+
Sauer et al. 2012 [45]	Education + Environmental Restructuring	Parents received written discharge information and access to a telephone hotline to assist with scheduling follow-up care in orthopedics.	Parent	Physicians	Reducing rates of ED use.	+
Scarfi et al. 2009 [83]	Education + Environmental Restructuring	Children in the intervention group received a skin allergen to determine allergens that could be linked to an asthma episode. Parents were provided with a copy of the allergen test to encourage follow-up care.	Patient and parent	Clinicians and administrator of the skin allergen test	Attending follow-up appointment.	+
Yin et al. 2008 [63]	Education + Enablement	Parents received illustrated resources about proper dosing of liquid medication. A research assistant reviewed resources with parents and had parent demonstrate how they would administer a medication dose.	Parent	Research team	Parent knowledge of medication dosing accuracy.	+
Zorc et al. 2009 [82]	Education + Environmental Restructuring	Parents watched a video and were mailed a letter to schedule follow-up care for their child’s asthma. Parents of children with persistent asthma also received an additional letter to encourage follow-up with a PCP.	Parent	Research team	Parent compliance with scheduling and attending follow-up appointment.	No change
Komoroski et al. 1996 [93]	Incentivization + Environmental Restructuring	Parents received one of two interventions. The first intervention group had their follow-up appointment booked for them and received a written reminder. The second intervention group received the same intervention as the first group with the addition of a mailed reminder one week prior to the appointment, a reminder phone call the day before the appointment, a work excuse, assistance with transportation to and from the appointment, and receiving child care.	Parent	Research team	Parent compliance with attending follow-up appointment.	+
3 Intervention functions						
Ducharme et al. 2011 [61]	Education + Enablement + Environmental Restructuring	Parents received a structured written action plan with information about asthma management, treatment, in addition to an asthma assessment tool and a prescription. A valved spacer and MDIS were also provided for children. Surveys and/or telephone calls were completed to determine parents’ completion of educational classes, follow-up visits with PCP and number of return visits to the ED.	Parent	ED physicians and pharmacists	Adherence to prescribed asthma medications four weeks following ED discharge.	+
Gorelick et al. 2006 [62]	Education + Enablement + Environmental Restructuring	Parents received one of two interventions. Group one received standardized information in addition to having information faxed to their PCP, and phone calls to offer assistance scheduling the follow-up care with PCP. The second group received the same care as intervention group one, in addition to being assigned a nurse or social worker to provide home visits, and additional education and links to community services.	Parent	Research team and home health care staff	Rates of return ED visits six months following initial ED asthma visit.	No change
Rotheram-Borus et al. 2000 [91]	Enablement + Training + Modeling	ED staff and health care providers received additional training surrounding mental health. Patients and parents were shown a video (20 min) in the ED about mental health treatments. Patients and parents also received therapy sessions, including identifying positive coping mechanisms, and outpatient treatments. Outpatient treatments included additional therapy sessions to use problem solving and roleplaying techniques to assist with family issues and future suicidal feelings.	Patient, parent and health care provider	Research team, ED staff and clinicians	Reducing suicidal behavior.	+
Smith et al. 2006 [90]	Enablement + Training + Incentivization	An asthma coach worked with parents to assist with their asthma concerns. Coaches also provided information about the importance of asthma follow-up care with a PCP and helped parents identify and address barriers to follow-up. Additionally, parents received a monetary incentive for attending follow-up care appointment after ED visit.	Parent	Asthma coach	Attending asthma planning visit with PCP.	No change
Sockrider et al. 2006 [89]	Education + Enablement + Environmental Restructuring	Asthma coaches utilized a computer-based resource that provided a customized written asthma action plan that was provided to parents and sent to their PCP. Asthma coaches conducted follow-up calls with parents to ensure follow-up with their PCP and to reinforce messages from the asthma plan. A phone line was also provided so parents could call with asthma management questions.	Health care provider and parent	Clinicians, respiratory care practitioners, and a layperson	Parent confidence managing asthma and reducing ED visits.	+
4 Intervention functions						
Asarnow et al. 2011 [92]	Education + Environmental Restructuring + Restriction + Training	Family members received education and training about the importance of mental health, outpatient treatment, how to provide support and ways to remove access to potential lethal means in the house. Patients and family members also received therapy sessions, including how to identify potential triggers and how to develop safe and healthier coping mechanisms for potential future suicidal thoughts.	Patient and parent	Clinicians	Rates of follow up outpatient treatment after ED discharge.	+

*ED* emergency department, *MDIS* metered-dose inhaler spacers, *PCP* primary care provider

### Quality of the evidence

Study quality was assessed using critical appraisal checklists from the Joanna Briggs Institute (JBI) [22]. The majority of the cross-sectional studies were of moderate quality (6/8 appraisal criteria present). The two appraisal questions that had mixed results were related to identifying and addressing potential confounding. Only a third of the studies (*n* = 9; 32%) identified possible confounders and even fewer studies noted how these confounders were adjusted for during data analysis (*n* = 8; 29%).

The 24 RCT were appraised using the Checklist for Randomized Controlled Trials and 46% of the studies (*n* = 11) did not clearly identify if a true randomization method was used. Additionally, only four RCT clearly stated how allocation concealment was used during the study [60–63].

The Checklist for Quasi-Experimental Studies was used for 11 studies in the review. These quasi-experimental studies (*n* = 11) scored consistently well across all of the appraisal checklist criteria, with the exception of concerns about composition of the comparison groups. We found that just over half of studies (*n* = 6; 55%) had at least one or more key variables with a 10% difference or more between the study groups. These differences raise some concerns about potential selection bias.

Finally, the three included qualitative studies were assessed using the Checklist for Qualitative Research. Deficits were identified in all studies regarding reporting of the philosophical perspective guiding the study methods and clearly describing the role of the researchers within the studies.

### Types of interventions

We identified 44 discharge communication interventions among the 75 included studies in the review (Table 2). Interventions were comprised of one to four intervention function types, according to the BCW (Table 3) [20]. The heterogeneity of intervention descriptions and outcome measures limited our ability to carry out meta-analysis.

**Table 3:** Behavior change wheel domains identified in intervention studies (*n* = 44)

Authors, publication year	Illness presentation	Identified BCW domains							Direction of Effect
		Education	Incentivization	Training	Enablement	Modeling	Environmental restructuring	Restriction	
Baker et al. 2009 [64]	Fever	✓							+
Bloch and Bloch 2013 [67]	Asthma, fever, vomiting, or diarrhea	✓							+
Boychuk et al. 2006 [70]	Asthma	✓							+
Brooks et al. 2017 [90]	Concussion	✓							+
Chande et al. 1996 [69]	Minor illnesses	✓							–
Cheng et al. 2002 [27]	Asthma	✓							+
Considine and Brennan 2007 [78]	Fever	✓							+
Delp and Jones 1996 [60]	Lacerations	✓							+
Hanson et al. 2017 [33]	Pain presentations	✓							+
Hart et al. 2015 [72]	Fever	✓							+
Gaucher et al. 2011 [32]	Various illnesses	✓							+
Isaacman et al. 1992 [74]	Otitis media	✓							+
Ismail et al. 2016 [68]	Fever and closed head injury	✓							+
Kaestli et al. 2015 [59]	Illnesses requiring prescription	✓							+
LeMay et al. 2010 [73]	Various injuries	✓							No change
Macy et al. 2011 [65]	Asthma	✓							+
Patel et al. 2009 [76]	Gastroenteritis	✓							+
Petersen et al. 1999 [77]	Asthma	✓							No change
Stevens et al. 2012 [66]	Pediatric pain	✓							+
Thomas et al. 2017 [51]	Mild traumatic brain injuries	✓							+
Waisman et al. 2005 [54]	Various illnesses	✓							+
Williams et al. 2013 [75]	Asthma	✓							+
Wood et al. 2017 [71]	Gastroenteritis, bronchiolitis, and fever	✓							+
Zorc et al. 2003 [82]	Asthma				✓				+
Hussain-Rizvi et al. 2009 [81]	Asthma					✓			+
Porter et al. 2006 [80]	Asthma						✓		No change
To et al. 2010 [79]	Asthma						✓		+/No change/No change
Cushman et al. 1991 [86]	Bike injury	✓			✓				No change
Jones et al. 1989 [84]	Otitis media	✓			✓				+
Pizarro et al. 1979 [43]	Diarrhea	✓			✓				+
Yin et al. 2008 [63]	Various illnesses	✓			✓				+
Kruesi et al. 1999 [87]	Mental health	✓					✓		+
Phillips 2009 [42]	Asthma	✓					✓		+
Sauer et al. 2012 [49]	Fractures	✓					✓		+
Scarfi et al. 2009 [83]	Asthma	✓					✓		+
Zorc et al. 2009 [85]	Asthma	✓					✓		No change
O’Neill-Murphy et al. 2001 [88]	Fever	✓				✓			+
Komoroski et al. 1996 [93]	Minor, acute infections		✓				✓		+
Ducharme et al. 2011 [61]	Asthma	✓			✓		✓		+
Gorelick et al. 2006 [62]	Asthma	✓			✓		✓		No change
Sockrider et al. 2006 [89]	Asthma	✓			✓		✓		+
Smith et al. 2006 [90]	Asthma		✓	✓	✓				No change
Rotheram-Borus et al. 2000 [91]	Mental health			✓	✓	✓			+
Asarnow et al. 2011 [92]	Mental health	✓		✓			✓	✓	+

#### One intervention function type

The majority of studies (27/44) leveraged a single intervention function type and focused on a range of illness presentations (Table 3). Twenty-three of these involved an *education* (sharing information) function and primarily focused on evaluating different modes of delivering information about an illness or instructions for managing care at home [27, 32, 33, 51, 54, 59, 60, 64–78, 94]. Almost half (10/23) of these studies examined the use of technology, including video [64–71] or interactive websites [72, 78], as education delivery systems. Technology-enabled tools were used as stand-alone delivery systems [64–67, 72] or enhanced with written and/or face-to-face interaction with staff [68–70]. All studies targeting parents in the ED had a primary goal to increase knowledge [65–68, 71, 72], adherence to guidelines [70], or reduce unnecessary ED return visits [69]. Only one technology-enabled *education* type intervention targeted healthcare providers and evaluated the effect of two 30-min online tutorials for ED nursing staff focused on physiology and management of fever and febrile convulsions on the discharge advice given to parents [78]. Overall, technology-enabled *education* type interventions targeting parents had a positive impact on knowledge acquisition and adherence to guidelines, but were not effective in reducing unnecessary return visits to the ED (Table 2).

The remaining *education* type intervention studies (13/23) examined printed discharge communication alone [73, 77, 94] or with other supports such as cartoons [33, 60], verbal reinforcement of written instructions [51, 54, 59, 74–76], confirming appropriate understanding through observation of parent technique [27], or verbal instructions alone [32]. These studies targeted a range of illness presentations and examined a variety of outcomes including feasibility in the ED, improvement of parent’s knowledge, comprehension and/or recall about specific diagnosis, important signs and symptoms or treatment instructions, and adherence to instructions (including follow-up). Overall direction of effect was positive in most studies examining parent recall of discharge information [33, 51, 76] and knowledge and comprehension [54, 59, 60] (Table 2).

The four single intervention studies that did not involve *education* as an intervention function contained *environmental restructuring* (changing the physical and social context) [79, 80], *modeling* (providing an example for people to aspire to or imitate) [81], or *enablement* (increasing the means/reducing barriers to increase capability or opportunity) [82]. Both *environmental restructuring* interventions targeted healthcare providers, focused on asthma, and included paper-based reminders. There was no significant change in provider behavior in either study (Table 2).

#### Two intervention function types

Eleven of the included studies combined two intervention functions in their discharge communication strategy (see Table 2). Again, *education* was a common intervention function across ten of these studies, with a second intervention function added to either overcome existing barriers (*environmental restructuring*) [42, 49, 83, 85, 87] or provide additional support to encourage a specific behavior (*enablement* [43, 63, 84, 86] or *modeling* [88]). Only one intervention did not include an education component, instead favoring *incentivization* and *environmental restructuring* [93]. All two intervention function studies targeted parents or parents/children and were generally focused on either improving care at home or improving adherence with follow-up instructions.

#### Three intervention function types

Five studies reported using a combination of three different intervention function types, including *education*, *enablement*, *environmental restructuring*, *training*, and *incentivization* [61, 62, 89–91] (Table 2). Four of the studies were focused on improving asthma management following an ED visit and one was focused on improving care provided to adolescent females who attempted suicide and presented to an ED. Three of the four asthma-focused studies used *education* in combination with *enablement* and *environmental restructuring* [61, 62, 89].

#### Four intervention function types

We identified one study that included four intervention functions (Table 3). Asarnow et al. (2011) developed an intervention which included a brief crisis therapy session in the ED and a structured telephone contact for motivating and supporting outpatient treatment within the first 48 h after the ED visit. We noted this intervention involved *education*, *environmental restructuring*, *restriction*, and *training* to improve follow-up rates of youth experiencing suicidality treated in an ED [92].

### Implementation strategies

The majority of interventions were delivered to children and parents by clinicians [27, 32, 42, 43, 49, 54, 60, 61, 67, 68, 71, 74, 76, 77, 81, 83, 86, 87, 89, 90, 92]. Various clinicians were involved in the implementation of interventions including ED clinicians [27, 42, 49, 54, 60, 61, 65, 68, 75, 80, 81, 83, 86, 87, 92], medical students/residents [67, 74], primary care providers (PCP) [82, 85], nurses [32, 43, 71, 84], pharmacists [61], and respiratory therapists [77]. Additionally, one study utilized a discharge facilitator to provide specific discharge communication to parents for gastroenteritis [76].

Most studies provided few details about the timing of intervention implementation, typically referencing that it was provided during the ED stay [60, 61, 63, 64, 66–68, 73, 74, 76, 80–92]. Thus, it was difficult to determine the exact point during the care process the intervention was delivered and the duration of the intervention. When specific timing of the interventions was indicated, it was either described as at the time of discharge [63], upon ED admission [77], directly following triage [65], or after the child was evaluated by an ED physician [69].

### Conceptual mapping

**Fig. 2:**
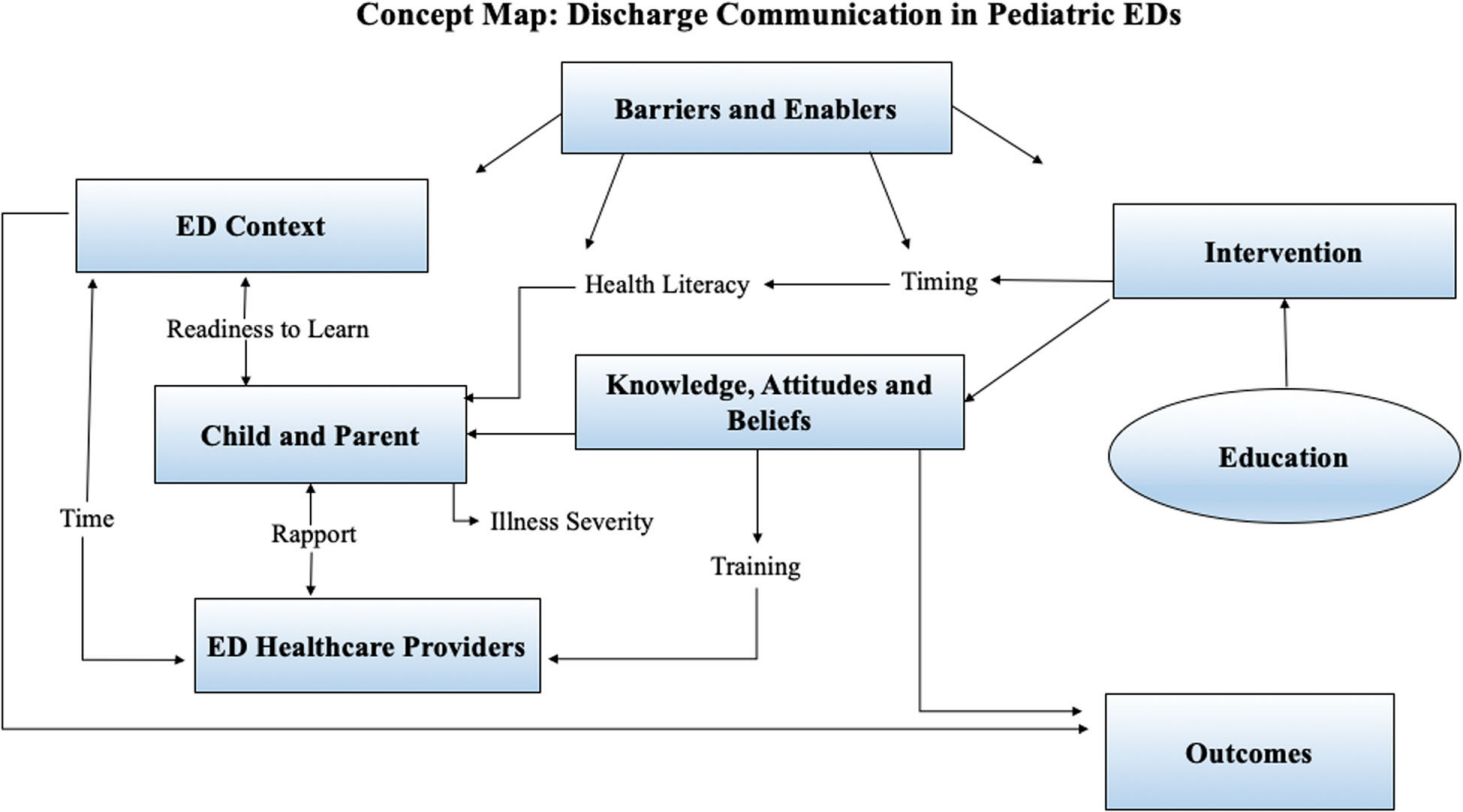
Concept Map

Conceptual mapping is a useful technique for exploring relationships in the data of narrative synthesis [98]. Using a consensus approach, three members of the research team reviewed the narrative summaries of all included studies during two half-day meetings and identified several key concepts and relevant terms to describe how discharge communication worked or did not work for children and their parents in ED settings (Fig. 2). The final schematic depicts the key barriers and enablers for discharge communication across all included studies. Key concepts include ED context, knowledge, attitudes and beliefs, ED healthcare providers, child and parents, intervention, education, and outcomes. Important linking terms include health literacy, timing, illness severity, readiness to learn, rapport, and training.

Barriers and enablers to successful discharge communication were identified at a number of levels, including the intervention, healthcare providers, child/parent, and the context in which care is delivered. The majority of interventions targeted parents and included education as a core strategy. Although not explicitly stated, the educational interventions in the studies appear to operate on the assumption that imparting information will improve health care provider or parent knowledge and subsequently change behaviors. Few studies used theory to guide design of the intervention [71, 80, 81, 83, 84, 89, 90, 95], and there was no formal assessment of potential barriers to changing parent, child, or health care provider behaviors. This made it difficult to understand how the interventions were expected to improve outcomes. Barriers related to intervention design (language, literacy level, content, readability) and delivery (timing during the visit, duration, fidelity, mode, extent of interactivity) were identified as important factors across a number of studies [26, 55, 57, 60, 65–67, 74, 78, 84, 86, 89, 99]. While there was variation in who delivered the interventions, it is important to highlight that in a number of the experimental trials, the interventions were delivered by individuals who were not regular members of the ED team [33, 51, 59, 63, 64, 66, 69, 70, 72, 73, 79, 82, 85, 88]. A collaborative approach to co-designing interventions was proposed by some authors as a possible strategy to enhance implementation [33, 40, 49, 70].

A number of barriers and enablers related to the patient and parent were reported including complexity of the patient presentation [53, 57, 69, 75, 84], first language and health literacy [35, 42, 46, 52], expectations of care [25], and past experience [66, 71]. Parents’ health literacy was considered an important factor in both the design [54, 63, 67] and the mode of delivery [46, 50, 57, 65] of the intervention. Parental stress was also considered an important influence on parents’ readiness to engage in discharge communication [26, 53, 69]. Actively engaging parents in the discharge process throughout their ED visit [27, 78, 81] and presenting information using multiple delivery modes [33, 51, 68, 75, 80, 83, 96] were thought to be important enablers. Parent decision making about the nuances of follow-up care post discharge can be complex [94]. It was also recognized that parents leverage a number of external knowledge sources including family and non-healthcare workers when making decisions about care for their child at home following an ED visit [52, 64, 65, 97].

Several studies suggested that ED healthcare providers recognize discharge communication as an important aspect of their everyday professional practice for both clinical and medicolegal reasons [23, 32], but there appears to be a fuzzy boundary between patient education and the provision of discharge information [70]. Discharge communication practice variation was common across a number of studies [30, 31, 100]. Healthcare provider training [23, 27, 28, 55, 74, 77, 86], beliefs and attitudes about discharge communication [40, 41, 57], and limited use of medical jargon [53, 96] were identified as important factors influencing success. Rapport between healthcare providers and parents was also considered an important factor contributing to successful discharge communication [69, 81, 95]. Although we did not identify any interventions that focused specifically on building rapport between providers and parents, the quality of the interaction between providers and patients/parents was highly valued as contributing to successful discharge communication [34, 46, 60, 69, 76, 81, 84, 88, 97].

The context of the ED including factors related to managing multiple patients in a compressed period of time and emphasis on patient flow can pose unique barriers to discharge communication [30, 55, 92]. Approaches to address some of these contextual barriers include a dedicated space for teaching, easily accessible resources, and the inclusion of strategies that reduce the time burden on ED providers and can easily be incorporated into the busy workflow of the ED [55, 79, 80, 86, 96]. However, Macy et al. (2011) noted that even the use of video education can be hindered during times of high patient volumes [65]. Ideal structures support discharge communication that is consistent and comprehensive yet succinct and relevant [23, 44, 97]. ED team practice norms regarding discharge communication [42, 78] and roles in the ED specifically dedicated to discharge communication may assist with consistent and reliable delivery [27, 39, 76].

Although there was wide variation in primary outcomes across studies, parent knowledge, comprehension, and recall were deemed important outcomes in a number of studies [33, 51, 59, 63, 65, 67, 68, 70–72]. Unfortunately, there was little consistency in how knowledge, comprehension, and recall were measured across these studies.

### Policies guiding pediatric discharge communication

We received a total of 36 responses to our email surveys (Canada = 17; Australia = 13, USA = 6) with no relevant pediatric ED discharge communication policies or guidelines identified. Following our search of the gray literature, three documents were discovered that included either policies or guidelines regarding the provision of discharge communication [101]. A joint policy developed by the American Academy of Pediatrics and the Committee on Pediatric Emergency Medicine recommended the provision of discharge communication within a family-centered care approach, [101]. Generally, this guideline contained very little direction in the way of discharge content and processes, and it is unclear if it translates into active policy development [101]. The Scottish Intercollegiate Guidelines Network (SIGN) has produced a generic discharge document tool that can be implemented to guide the discharge process [102]. The discharge document provides guidance on important content that should be communicated at discharge, including primary discharge diagnosis, presenting complaint, significant procedures, new medications, and follow-up instructions [102]. However, no specific recommendations are made about how the SIGN discharge document should be generated, updated, and used in ED clinical practice [102]. Finally, the National Guidelines Clearinghouse contained one active policy that is currently utilized by affiliated children’s hospitals in the USA. This policy outlines standards for discharge communication as the primary responsibility of the ED nurse and that all patients are to be discharged home with explicit plans for follow-up care, a summary of the visit, appropriate instructions for care, and prescription medications [103]. Prior to discharge, it is the responsibility of the nurse to verify that all health care concerns have been addressed, parents are aware of when to return to the ED, documentation is complete, and medications have been reviewed with the patient [103].

## Discussion

The body of literature exploring discharge communication in a pediatric ED context is highly heterogeneous, reporting a range of interventions, delivery methods, and outcome measures across a variety of illness presentations. These findings are consistent with systematic reviews of discharge communication practices in hospital settings [15, 104]. In general, our findings demonstrate that discharge communication in pediatric emergency practice environments has been largely oversimplified with a corresponding atheoretical approach to intervention design, involving strategies that ignore the context of where the communication takes place (ED) and where the information is primarily used by the parents (home). Education was the most common type of intervention evaluated. The underlying assumption appears to be that a parent’s lack of knowledge is the most important contributor to poor child outcomes and educational interventions are the most viable solution. We noted a lack of focus on child and youth comprehension and limited attention on healthcare provider knowledge and skills to deliver discharge communication. However, through a concept mapping exercise, we were able to identify factors at the individual, intervention, and system level that may affect successful discharge communication in a pediatric emergency care context.

Many of the educational interventions included in our review relied on passive dissemination strategies to deliver information. Yet, we know that behavior change requires more than simply the acquisition of knowledge [105]. Passive dissemination of educational material is rarely effective in changing the behavior of providers or improving patient outcomes [106, 107]. Adults learn best when there is a perceived need, active participation, reinforcement of new behaviors, and immediate feedback and correction of misconceptions [108, 109]. Active engagement in learning such as the use of teach-back strategies have been associated with improved comprehension, more patient-centered communication, and increased engagement of parents [110–112]. Teach-back is a way of checking comprehension by asking patients to repeat back in their own words what they have been told by their health care provider [113]. However, further research is needed regarding the efficacy, feasibility, fidelity and acceptability of teach-back in an ED setting [112]. The use of behavior change theories to specify behavioral targets and guide intervention design in future discharge communication studies are needed to strengthen this body of literature and assist with identifying and evaluating the “active ingredients” that affect intervention outcomes [114].

While standardizing discharge communication may be viewed as one way of achieving good quality discharge communication, our synthesis demonstrated conflicting results pertaining to the effectiveness of standardized discharge communication. Standardized instructions are often recommended to increase familiarity with ED discharge documentation for patients, staff, and general practitioners [115]. One study in this synthesis showed improved communication of important discharge information through the use of standardized discharge communication in children with otitis media [84]. However, other authors reported that standardized instructions were insufficient to enhance adherence and comprehension [50]. In light of this, ensuring that discharge instructions contain common elements, while still allowing for adaptation and tailoring to accommodate provider and parent needs, might be a more appropriate approach.

Finally, our findings reveal a dearth of existing policies guiding discharge communication practice in a Canadian pediatric ED context. Further, there appears to be a lack of clarity regarding professional accountability for discharge communication in the literature. Tavender et al. (2014) examined factors that influence management of mild traumatic brain injury in the ED. They noted that physicians felt primarily responsible for discharge communication as they were the health care provider responsible for discharging a patient; however, nurses felt discharge communication should be a shared responsibility to ensure patients were adequately educated prior to being sent home [116]. Many of the studies included in our review, particularly those related to asthma [79], recognized the importance of an interdisciplinary approach to discharge communication. Clear guidelines and polices regarding discharge communication practice are critical to ensure more sustainable and reliable discharge communication strategies in the future.

### Strengths and limitations

This review followed established review methodologies including a comprehensive search of the published and gray literature, assessment of risk of bias, and established narrative synthesis strategies to explore relationships across a range of study designs. We incorporated an iKT approach to ensure our findings were relevant to stakeholder needs.

Due to the heterogeneity of the interventions and outcomes, we were unable to perform a meta-analysis. While we used an established behavioral framework to guide our examination of discharge communication interventions, our classification of the included interventions was limited to the details provided in the study reports. It is possible that the interventions contained additional functions that were not captured in our analysis because they were not reported by the study author.

## Conclusion

Improving discharge communication for parents in an ED setting presents a significant opportunity for improving health outcomes for children. The majority of existing strategies to improve discharge communication have been educational strategies targeting parents. Furthermore, theory-based interventions are rare, making it difficult to discern the active ingredients underlying successful interventions. Findings from this review highlight a number of opportunities for ED researchers, clinicians, administrators, and decision-makers to consider strengthening discharge communication policies and improve the design of discharge communication interventions. Effective discharge communication strategies in a pediatric emergency practice context can improve parent comprehension, increase adherence to treatment plans, reduce unnecessary return visits, optimizing health system use, and improving health outcomes for children.

## Additional files

Additional file 1: Search Strategy Terms. (PDF 55 kb)

Additional file 2: Inclusion and Exclusion Criteria. (ZIP 38 kb)

Additional file 3: List of Excluded Studies. (XLS 95 kb)

## Abbreviations

ARCHE: Alberta Research Centre for Child Health Evidence
BCW: Behavior Change Wheel
CI: Confidence interval
ED: Emergency department
HBM: Health belief model
iKT: Integrated knowledge translation
JBI: Joanna Briggs Institute
MDIS: Metered-Dose Inhaler Spacers
NAEEP: National Asthma Education and Prevention Program
PCP: Primary care provider
PECARN: Pediatric Emergency Care Applied Research Network
PEM-CRC: Pediatric Emergency Medicine Collaborative Research Committee
PERC: Pediatric Emergency Research Canada
PERUKI: Pediatric Emergency Research in the United Kingdom & Ireland
PREDICT: Pediatric Research in Emergency Departments International Collaborative
RCT: Randomized controlled trial
SIGN: Scottish Intercollegiate Guidelines Network
