# Supplementary material for: Discharge communication practices in pediatric emergency care: a systematic review and narrative synthesis
Source: Syst Rev. 2019 Apr 3;8:83. doi: 10.1186/s13643-019-0995-7 (PMC6446263; doi:10.1186/s13643-019-0995-7)
Supplement: Supplementary file 1 — Search Strategy Terms. (PDF 55 kb) [file 13643_2019_995_MOESM1_ESM.pdf]

Emergency Setting:
1. ((Emergen* or urgen* or critical) adj3 (service* or care or department* or centre* or center* or room*)).tw.
2. exp emergency service, hospital/
Child/Parent Population:
3. exp child/
4. exp infant/
5. adolescent/
6. exp parents/
7. exp legal guardians/
8. infan*.tw.
9. child*.tw.
10. preschool.tw.
11. (babies or baby).tw.
12. neonat*.tw.
13. youth.tw.
14. young.tw.
15. parent*.tw.
16. p?ediatr*.tw.
17. teen*.tw.
Terms for Discharge
18. patient discharge/
19. patient transfer/
20. convalescence/
21. discharg*.tw.
22. transfer*.tw.
23. releas*.tw.
24. follow up or followup.tw.
25. Follow-Up Studies/
26. aftercare/
Instructions Interventions:
27. education/
28. Patient education as topic/
29. patient education handout.pt.
30. self care/
31. "models, educational"/
32. pamphlets/
33. continuity of care/
34. education.tw.
35. (discharg* adj3 (information or advice or educat*)).tw.
36. ((patient* or carer* or parent*) adj3 information).tw.
37. ((patient* or carer* or parent*) adj3 educat*).tw.
38. continuity of patient care/
39. (continuity adj3 care).tw.
40. medical information.tw.
41. written information.tw.

42. pamphlet*.tw.
43. (booklet* or brochure* or leaflet$ or postcard*).tw.
44. 1 or 2
45. or/3-17
46. or/18-26
47. or/27-43
48. 44 and 45
49. 48 and 46
50. 49 and 47

Other possible search terms for the emergency care setting:
Emergencies/
Emergency Medical Technicians/
Emergency Medicine/
Emergency Treatment/
exp Emergency Medical Services/
Outpatient Clinics, Hospital/
Outpatients/
((accident* or casualt*) adj3 (service* or department* or room* or center* or centre* or clinic or clinics or unit*)).tw.
((acute or emerg*) adj3 care).tw.
(ED or EDs or ER or ERs or EMS).tw.
(emerg* adj3 (department* or room* or ward* or unit* or hospital* or clinic or clinics or patient* or physician* or doctor* or treatment*)).tw.
(emerg* adj3 (technician* or practitioner* or dispatch*)).tw.
(emergicenter* or emergicentre*).tw.
((out-patient* or outpatient*) adj3 (service* or department* or room* or center* or centre* or clinic or clinics or unit*)).tw.
((prehospital or pre-hospital) adj3 (emergenc* or care*)).tw.
(primary adj3 care).tw.
(trauma adj3 (center* or centre* or clinic or clinics)).tw.
(urgent adj3 care).tw.
((walkin or walk-in) adj3 (service* or department* or room* or center* or centre* or clinic or clinics or unit*)).tw.
