# Supplementary material for: Discharge communication practices in pediatric emergency care: a systematic review and narrative synthesis
Source: Syst Rev. 2019 Apr 3;8:83. doi: 10.1186/s13643-019-0995-7 (PMC6446263; doi:10.1186/s13643-019-0995-7)
Supplement: Supplementary file 2 — Inclusion and Exclusion Criteria. (ZIP 38 kb) [file 13643_2019_995_MOESM2_ESM.zip › Additional file 2_inclusion and exclusion criteriaR1.docx]

Inclusion and Exclusion Criteria

| **Inclusion** | **Exclusion** |
| --- | --- |
| - Children ages 0-19 years old - Children presenting to the ED with an urgent illness presentation - Primary objective(s) relates to elements and/or content related to discharge communication processes - If primary outcome data includes children and adult content, children data must be presenting separately | - Studies focuses on adults - Studies conducted outside of an ED setting - Parent and child data is not presented separately - Primary outcomes not related to discharge communication |
